# (3*E*,5*E*)-1-Benzyl-3,5-bis­(2-fluoro­benzyl­idene)piperidin-4-one

**DOI:** 10.1107/S1600536809039609

**Published:** 2009-10-07

**Authors:** R. S. Rathore, N. S. Karthikeyan, K. Sathiyanarayanan, P. G. Aravindan

**Affiliations:** a Bioinformatics Infrastructure Facility, Department of Biotechnology, School of Life Science, University of Hyderabad, Hyderabad 500 046, India; b Chemistry Division, School of Science and Humanities, VIT University, Vellore 632 014, India; c Physics Division, School of Science and Humanities, VIT University, Vellore 632 014, India

## Abstract

The inversion-related mol­ecules of the title compound, C_26_H_21_F_2_NO, associate into closed dimeric subunits *via* co-operative C—H⋯π inter­actions. Two non-classical C—H⋯O and one C—H⋯N intra­molecular hydrogen bonds are also found in the crystal structure. The piperidin-4-one ring adopts a sofa conforamtion with the 1-benzyl group in the equatorial position, and the equiplanar fluoro­phenyl substituents in the 3- and 5-positions stretched out on either side. The 1-benzyl group is disposed towards the substituent in the 6th position of the piperidin-4-one ring. The 3,5-diene units possess *E* configurations.

## Related literature

For the synthesis of and pharmaceutical studies on 3,5-diaryl­idene-4-piperidone compounds, see: Krapcho & Turk (1979 ▶); Das *et al.* (2007 ▶). For a related structure, see: Suresh *et al.* (2007 ▶). For ring conformations, see: Cremer & Pople (1975 ▶), Duax *et al.*, (1976 ▶). For C—H⋯π inter­actions, see: Nishio *et al.* (2009 ▶).
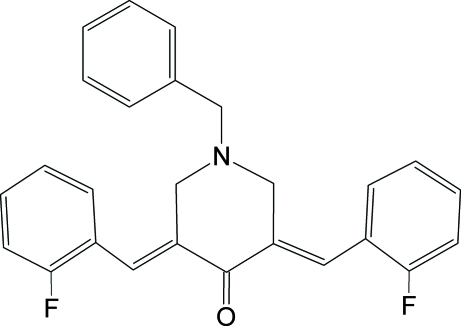


## Experimental

### 

#### Crystal data

C_26_H_21_F_2_NO
*M* *_r_* = 401.44
Triclinic, 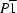

*a* = 6.7738 (4) Å
*b* = 12.5652 (7) Å
*c* = 12.8535 (7) Å
α = 71.051 (1)°
β = 88.057 (2)°
γ = 89.117 (2)°
*V* = 1034.12 (10) Å^3^
*Z* = 2
Mo *K*α radiation
μ = 0.09 mm^−1^
*T* = 298 K
0.19 × 0.18 × 0.12 mm

#### Data collection

Bruker APEXII CCD area-detector diffractometer
Absorption correction: multi-scan (*SADABS*; Bruker, 2004 ▶) *T*_min_ = 0.966, *T*_max_ = 0.984
13326 measured reflections
4281 independent reflections
2531 reflections with *I* > 2σ(*I*)
*R*_int_ = 0.029

#### Refinement

*R*[*F* ^2^ > 2σ(*F* ^2^)] = 0.048
*wR*(*F* ^2^) = 0.135
*S* = 1.12
4281 reflections
271 parameters
H-atom parameters constrained
Δρ_max_ = 0.16 e Å^−3^
Δρ_min_ = −0.15 e Å^−3^

### 

Data collection: *APEX2* (Bruker, 2004 ▶); cell refinement: *SAINT-Plus* (Bruker, 2004 ▶); data reduction: *SAINT-Plus*; program(s) used to solve structure: *SHELXS97* (Sheldrick, 2008 ▶); program(s) used to refine structure: *SHELXL97* (Sheldrick, 2008 ▶); molecular graphics: *ORTEP-3* (Farrugia, 1997 ▶); software used to prepare material for publication: *PLATON* (Spek, 2009 ▶).

## Supplementary Material

Crystal structure: contains datablocks global, I. DOI: 10.1107/S1600536809039609/rk2161sup1.cif

Structure factors: contains datablocks I. DOI: 10.1107/S1600536809039609/rk2161Isup2.hkl

Additional supplementary materials:  crystallographic information; 3D view; checkCIF report

## Figures and Tables

**Table 1:** Hydrogen-bond geometry (Å, °)

*D*—H⋯*A*	*D*—H	H⋯*A*	*D*⋯*A*	*D*—H⋯*A*
C14—H14⋯O1	0.93	2.40	2.772 (2)	104
C21—H21⋯O1	0.93	2.39	2.768 (2)	104
C7—H7*B*⋯*Cg*2^i^	0.97	2.78	3.7315 (19)	168
C13—H13⋯N1	0.93	2.56	2.873 (3)	100

Symmetry code: (i) 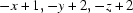. *Cg*2 is the centroid of the C8–C13 ring.

**Fig. 1.**
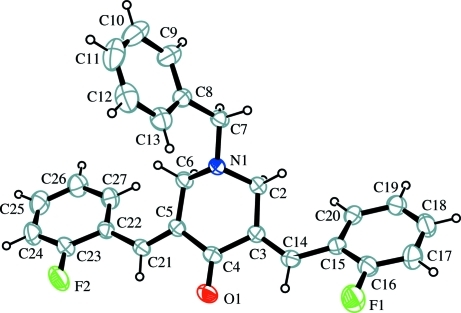
A view of I with the atom numbering scheme. Displacement ellipsoids are drawn at 30% probability level. H atoms are presented as a small spheres of arbitrary radius.

**Fig. 2.**
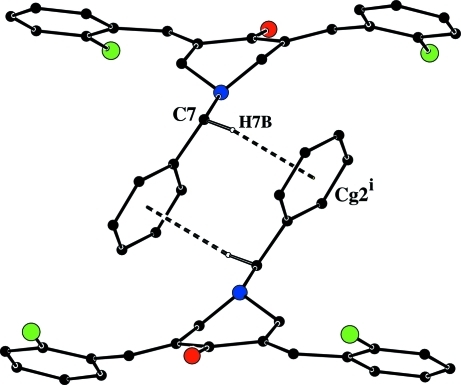
Molecular associations into closed dimers via cooperative C–H···π interactions (see Table 1 for symmetry code). Cg2 is the centroid of (C8-C13) ring.

## supplementary crystallographic information

### Comment

Derivatives of 3,5-diarylidene-4-piperidones (*D4P*) are pharmaceutically important compounds (Krapcho & Turk, 1979; Das *et al.*, 2007). During our investigations on *D4P*, a series of compounds were prepared. The molecular and crystal structure of title compound (3E,5E)-1-benzyl-3,5-bis[(2-fluorophenyl)methylidene]piperidin-4-one, (I), is reported here.

The molecular structure of I with atom numbering scheme is shown in Fig. 1. The 3,5-diene moieties possess *E*-configuration. The 3,5-difluorophenyl substituents of the piperidinone ring are stretched out on either side with following values of torsion angles: C4–C3–C14–C15 = 175.34 (16)°, C3–C14–C15–C16 = 147.49 (19)°, C4–C5–C21–C22 = -172.85 (15)°, and C5–C21–C22–C23 = -151.13 (17)°. The dihedral angle of 3,5-difluorophenyl units is 3.29 (7)°. The dihedral angles between of benzene rings of 3- and 5-substitutens with respect to the corresponding ring of 1-benzyl substituent are 58.65 (7)° and 56.90 (7)°, respectively.

The *sp*^2^ hybridized C3, C4 and C5 atoms give rise to a *sofa*-conformation of the six-membered piperidinone ring as also observed in the structures of related compounds, namely, (*R*)-3,5-Bis[(*E*)-benzylidene]-1-(1-phenylethyl)piperidin-4-one, 3,5-bis[(*E*)-4-chlorobenzylidene]-1-[(*R*)-1-phenylethyl] piperidin-4-one, and 3,5-bis[(*E*)-2-chlorobenzylidene]-1-[(*R*)-1-phenylethyl] piperidin-4-one (Suresh *et al.*, 2007). In the sofa conformation, the N1 atom is -0.781 (1)Å shifted out of the base plane (C2/C3/C4/C5/C6). The deviation of the ring from ideal *sofa*-conformation, ΔC_2_ (Duax *et al.*, 1976) is 3.4°. The Cremer and Pople (Cremer & Pople, 1975) puckering parameters, corresponding to the ring conformation are as follows: q2 = 0.5432 (16)Å, q3 = 0.2577 (17)Å, φ = 3.14 (18)°, θ = 64.62 (16)°, and total puckering amplitude Q = 0.6012 (16)Å. The benzyl substituent is in equatorial position of piperidinone ring and its conformation is described by the following torsion angles: C2–N1–C7–C8 = -162.82 (14)° and N1–C7–C8–C9 = -153.28 (15)°. The N1-benzyl group is disposed towards C6 substituent of the piperidin-4-one ring, a feature that varies among related structures.

The observed inter- and intra-molecular interactions are listed in Table 1. The adjacent H14 and H21 atoms participate in intra-molecular C14–H14···O1···H21–C21 interaction scheme. The crystal packing is characterized by C–H···π hydrogen-bonded dimers. The methylene and aromatic groups of the N1-benzyl substituent participate in the interaction forming C7–H7B···*Cg*2^i^ with symmetry code: (i) -*x*+1, -*y*+2, -*z*+2. The *Cg*2 is the centroid of (C8-C13) ring. The observed geometry of C–H···π interaction in I is in the range, reported by the Nishio and coworkers (Nishio *et al.*, 2009). Crystal packing is shown in Fig. 2.

### Experimental

A mixture of 1-benzyl-4-piperidone (0.01 mol) and 2-fluorobenzaldehyde (0.02 mol) was added to a warm solution of ammonium acetate (0.01 mol) in absolute ethanol (15 ml). The mixture was gradually warmed on a water bath until the yellow color changed to orange. The mixture was kept aside overnight at room temperature. Reactions were monitored with *TLC* for completeness. The solid obtained was separated and the crude compound were purified using silica gel column chromatography with hexane and ethyl acetate as elutant. Final yields: 96.19%; m.p. 415 (2)K. Suitable single crystals for data collection were grown from ethanol, tetrahydrofurane and benzene in (1:1:1) ratio.

### Refinement

Hydrogen atoms were placed in the geometrically expected positions and refined with the riding options. The distances with hydrogen atoms are: C(aromatic)–H = 0.93 Å, *C*(methylene)–H = 0.97 Å, and *U*_iso_(H) = 1.2*U*_eq_(C).

### Crystal data

**Table:**

C_26_H_21_F_2_NO	*Z* = 2
*M**_r_* = 401.44	*F*(000) = 420
Triclinic, *P*1	*D*_x_ = 1.289 Mg m^−^^3^
Hall symbol: -P 1	Melting point: 415(2) K
*a* = 6.7738 (4) Å	Mo *K*α radiation, λ = 0.71073 Å
*b* = 12.5652 (7) Å	Cell parameters from 3075 reflections
*c* = 12.8535 (7) Å	θ = 2.8–21.7°
α = 71.051 (1)°	µ = 0.09 mm^−^^1^
β = 88.057 (2)°	*T* = 298 K
γ = 89.117 (2)°	Block, colourless
*V* = 1034.12 (10) Å^3^	0.19 × 0.18 × 0.12 mm

### Data collection

**Table:**

Bruker APEXII CCD area-detector diffractometer	4281 independent reflections
Radiation source: fine-focus sealed tube	2531 reflections with *I* > 2σ(*I*)
graphite	*R*_int_ = 0.029
φ and ω scans	θ_max_ = 26.9°, θ_min_ = 2.0°
Absorption correction: multi-scan (*SADABS*; Bruker, 2004)	*h* = −6→8
*T*_min_ = 0.966, *T*_max_ = 0.984	*k* = −15→15
13326 measured reflections	*l* = −14→16

### Refinement

**Table:**

Refinement on *F*^2^	Primary atom site location: structure-invariant direct methods
Least-squares matrix: full	Secondary atom site location: difference Fourier map
*R*[*F*^2^ > 2σ(*F*^2^)] = 0.048	Hydrogen site location: inferred from neighbouring sites
*wR*(*F*^2^) = 0.135	H-atom parameters constrained
*S* = 1.12	*w* = 1/[σ^2^(*F*_o_^2^) + (0.0626*P*)^2^] where *P* = (*F*_o_^2^ + 2*F*_c_^2^)/3
4281 reflections	(Δ/σ)_max_ < 0.001
271 parameters	Δρ_max_ = 0.16 e Å^−^^3^
0 restraints	Δρ_min_ = −0.15 e Å^−^^3^

### Special details

**Table:**

Geometry. All s.u.'s (except the s.u. in the dihedral angle between two l.s. planes) are estimated using the full covariance matrix. The cell s.u.'s are taken into account individually in the estimation of s.u.'s in distances, angles and torsion angles; correlations between s.u.'s in cell parameters are only used when they are defined by crystal symmetry. An approximate (isotropic) treatment of cell s.u.'s is used for estimating s.u.'s involving l.s. planes.===========================================================================Weighted least-squares planes through the starred atoms (Nardelli, Musatti, Domiano & Andreetti Ric.Sci.(1965),15(II–A),807). Equation of the plane: m1**X*+m2*Y+m3**Z*=d Plane 1 m1 = 0.3436 (10) m2 = -0.8917 (5) m3 = -0.2946 (7) D = -14.293 (8) Atom d s d/s (d/s)**2 C2 * 0.0169 0.0018 9.390 88.168 C3 * -0.0140 0.0017 -8.219 67.556 C5 * 0.0135 0.0017 8.052 64.834 C6 * -0.0165 0.0018 -9.252 85.593 C4 -0.1534 0.0018 -84.823 7194.988 N1 -0.7455 0.0014 -534.558 285752.750 O1 -0.3305 0.0015 -221.129 48897.992 ============ Sum((d/s)**2) for starred atoms 306.151 Chi-squared at 95% for 1 degrees of freedom: 3.84 The group of atoms deviates significantly from planarity Plane 2 m1 = 0.3043 (8) m2 = -0.9090 (3) m3 = -0.2848 (7) D = -14.485 (7) Atom d s d/s (d/s)**2 C2 * -0.0011 0.0018 -0.607 0.368 C3 * 0.0338 0.0017 19.758 390.365 C5 * 0.0610 0.0017 36.177 1308.754 C6 * -0.0350 0.0018 -19.553 382.330 C4 * -0.0718 0.0018 -39.539 1563.307 N1 -0.7813 0.0014 -557.032 310284.906 O1 -0.1954 0.0015 -129.768 16839.834 ============ Sum((d/s)**2) for starred atoms 3645.124 Chi-squared at 95% for 2 degrees of freedom: 5.99 The group of atoms deviates significantly from planarity Plane 3 m1 = -0.3826 (9) m2 = -0.4121 (9) m3 = -0.8269 (6) D = -16.581 (12) Atom d s d/s (d/s)**2 C8 * -0.0025 0.0018 -1.437 2.066 C9 * 0.0039 0.0022 1.780 3.167 C10 * -0.0027 0.0028 -0.947 0.896 C11 * -0.0017 0.0033 -0.509 0.259 C12 * 0.0014 0.0030 0.464 0.215 C13 * 0.0014 0.0021 0.691 0.478 C7 -0.1040 0.0017 -60.838 3701.229 ============ Sum((d/s)**2) for starred atoms 7.082 Chi-squared at 95% for 3 degrees of freedom: 7.81 The group of atoms does not deviate significantly from planarity Plane 4 m1 = -0.2708 (9) m2 = -0.9623 (2) m3 = -0.0242 (8) D = -12.113 (12) Atom d s d/s (d/s)**2 C15 * -0.0045 0.0019 -2.435 5.931 C16 * 0.0046 0.0022 2.062 4.253 C17 * 0.0018 0.0025 0.733 0.538 C18 * -0.0063 0.0024 -2.623 6.878 C19 * 0.0030 0.0021 1.381 1.908 C20 * 0.0019 0.0019 1.030 1.061 F1 0.0148 0.0017 8.905 79.302 C14 0.0542 0.0019 29.279 857.250 ============ Sum((d/s)**2) for starred atoms 20.569 Chi-squared at 95% for 3 degrees of freedom: 7.81 The group of atoms deviates significantly from planarity Plane 5 m1 = -0.2350 (1) m2 = -0.9696 (2) m3 = -0.0685 (8) D = -12.925 (6) Atom d s d/s (d/s)**2 C22 * 0.0014 0.0018 0.732 0.536 C23 * -0.0006 0.0021 -0.301 0.091 C24 * -0.0019 0.0024 -0.785 0.616 C25 * 0.0034 0.0025 1.368 1.872 C26 * -0.0015 0.0024 -0.645 0.416 C27 * -0.0009 0.0021 -0.411 0.169 F1 0.0249 0.0017 14.969 224.077 C21 0.0995 0.0018 54.152 2932.435 ============ Sum((d/s)**2) for starred atoms 3.699 Chi-squared at 95% for 3 degrees of freedom: 7.81 The group of atoms does not deviate significantly from planarity Dihedral angles formed by LSQ-planes Plane - plane angle (s.u.) angle (s.u.) 1 2 2.53 (7) 177.47 (7) 1 3 61.34 (7) 118.66 (7) 1 4 39.45 (7) 140.55 (7) 1 5 36.49 (8) 143.51 (8) 2 3 60.42 (7) 119.58 (7) 2 4 36.94 (7) 143.06 (7) 2 5 33.97 (7) 146.03 (7) 3 4 58.65 (7) 121.35 (7) 3 5 56.90 (7) 123.10 (7) 4 5 3.29 (7) 176.71 (7)===========================================================================
Refinement. Refinement of *F*^2^ against ALL reflections. The weighted *R*-factor *wR* and goodness of fit *S* are based on *F*^2^, conventional *R*-factors *R* are based on *F*, with *F* set to zero for negative *F*^2^. The threshold expression of *F*^2^ > σ(*F*^2^) is used only for calculating *R*-factors(gt) *etc*. and is not relevant to the choice of reflections for refinement. *R*-factors based on *F*^2^ are statistically about twice as large as those based on *F*, and *R*-factors based on ALL data will be even larger.

### Fractional atomic coordinates and isotropic or equivalent isotropic displacement parameters (Å^2^)

**Table:**

	*x*	*y*	*z*	*U*_iso_*/*U*_eq_	
N1	0.27851 (17)	0.81199 (11)	0.92961 (10)	0.0473 (4)	
O1	−0.20803 (17)	0.63605 (12)	0.98723 (10)	0.0733 (4)	
F1	−0.02954 (18)	0.50536 (13)	1.36027 (10)	0.1039 (5)	
F2	−0.42414 (16)	0.85416 (12)	0.63705 (10)	0.1014 (5)	
C2	0.3146 (2)	0.70072 (14)	1.00807 (14)	0.0513 (4)	
H2A	0.3789	0.7077	1.0718	0.062*	
H2B	0.4009	0.6580	0.9744	0.062*	
C3	0.1218 (2)	0.64096 (13)	1.04272 (14)	0.0460 (4)	
C4	−0.0404 (2)	0.67177 (14)	0.96139 (14)	0.0504 (4)	
C5	0.0152 (2)	0.74206 (13)	0.84662 (13)	0.0450 (4)	
C6	0.2136 (2)	0.79813 (14)	0.82805 (13)	0.0494 (4)	
H6A	0.3090	0.7528	0.8029	0.059*	
H6B	0.2050	0.8711	0.7715	0.059*	
C7	0.4527 (2)	0.88369 (14)	0.91102 (14)	0.0531 (4)	
H7A	0.5473	0.8608	0.8639	0.064*	
H7B	0.5140	0.8729	0.9809	0.064*	
C8	0.4052 (3)	1.00649 (15)	0.85878 (14)	0.0534 (5)	
C9	0.5456 (3)	1.07860 (18)	0.79602 (17)	0.0773 (6)	
H9	0.6674	1.0505	0.7812	0.093*	
C10	0.5070 (6)	1.1944 (2)	0.7540 (2)	0.1077 (9)	
H10	0.6032	1.2432	0.7120	0.129*	
C11	0.3284 (7)	1.2350 (2)	0.7748 (2)	0.1146 (11)	
H11	0.3019	1.3117	0.7469	0.138*	
C12	0.1887 (4)	1.1639 (2)	0.8362 (2)	0.1007 (8)	
H12	0.0666	1.1921	0.8501	0.121*	
C13	0.2261 (3)	1.05075 (17)	0.87788 (17)	0.0713 (6)	
H13	0.1285	1.0030	0.9198	0.086*	
C14	0.0847 (2)	0.56488 (14)	1.14056 (15)	0.0548 (5)	
H14	−0.0447	0.5392	1.1546	0.066*	
C15	0.2232 (2)	0.51659 (14)	1.22897 (15)	0.0524 (4)	
C16	0.1619 (3)	0.48556 (17)	1.33750 (17)	0.0663 (5)	
C17	0.2810 (4)	0.43617 (19)	1.42435 (18)	0.0837 (6)	
H17	0.2319	0.4170	1.4965	0.100*	
C18	0.4741 (3)	0.41584 (18)	1.40188 (19)	0.0804 (6)	
H18	0.5586	0.3832	1.4593	0.096*	
C19	0.5436 (3)	0.44358 (16)	1.29479 (19)	0.0715 (6)	
H19	0.6745	0.4287	1.2798	0.086*	
C20	0.4199 (3)	0.49336 (14)	1.20967 (16)	0.0619 (5)	
H20	0.4689	0.5119	1.1376	0.074*	
C21	−0.1155 (2)	0.75128 (14)	0.76848 (14)	0.0498 (4)	
H21	−0.2389	0.7199	0.7930	0.060*	
C22	−0.0894 (2)	0.80438 (14)	0.64938 (14)	0.0509 (4)	
C23	−0.2488 (3)	0.85146 (16)	0.58508 (16)	0.0650 (5)	
C24	−0.2382 (4)	0.89583 (18)	0.47290 (19)	0.0832 (7)	
H24	−0.3495	0.9267	0.4335	0.100*	
C25	−0.0606 (4)	0.89395 (19)	0.41964 (18)	0.0851 (7)	
H25	−0.0505	0.9232	0.3433	0.102*	
C26	0.1030 (3)	0.84889 (18)	0.47902 (17)	0.0801 (6)	
H26	0.2240	0.8482	0.4428	0.096*	
C27	0.0879 (3)	0.80488 (16)	0.59185 (15)	0.0637 (5)	
H27	0.2000	0.7745	0.6308	0.076*	

### Atomic displacement parameters (Å^2^)

**Table:**

	*U*^11^	*U*^22^	*U*^33^	*U*^12^	*U*^13^	*U*^23^
N1	0.0468 (7)	0.0487 (8)	0.0461 (8)	−0.0052 (6)	−0.0086 (6)	−0.0143 (7)
O1	0.0423 (7)	0.1033 (11)	0.0658 (9)	−0.0083 (7)	−0.0004 (6)	−0.0156 (8)
F1	0.0798 (8)	0.1567 (13)	0.0676 (8)	0.0106 (8)	0.0028 (6)	−0.0270 (8)
F2	0.0582 (7)	0.1426 (12)	0.0905 (9)	0.0144 (7)	−0.0214 (7)	−0.0189 (8)
C2	0.0456 (9)	0.0543 (11)	0.0528 (10)	−0.0003 (8)	−0.0086 (8)	−0.0152 (9)
C3	0.0440 (9)	0.0485 (10)	0.0473 (10)	0.0010 (7)	−0.0008 (7)	−0.0181 (8)
C4	0.0390 (9)	0.0567 (11)	0.0585 (12)	0.0004 (8)	−0.0001 (8)	−0.0231 (9)
C5	0.0413 (9)	0.0474 (10)	0.0502 (10)	0.0045 (7)	−0.0034 (7)	−0.0211 (8)
C6	0.0479 (9)	0.0542 (10)	0.0485 (10)	−0.0024 (8)	−0.0053 (7)	−0.0197 (8)
C7	0.0497 (9)	0.0575 (11)	0.0553 (11)	−0.0058 (8)	−0.0064 (8)	−0.0219 (9)
C8	0.0678 (12)	0.0523 (11)	0.0451 (10)	−0.0096 (9)	−0.0064 (9)	−0.0218 (9)
C9	0.1022 (15)	0.0686 (15)	0.0628 (13)	−0.0219 (12)	0.0098 (12)	−0.0236 (11)
C10	0.180 (3)	0.073 (2)	0.0675 (16)	−0.0447 (19)	0.0029 (18)	−0.0176 (14)
C11	0.200 (3)	0.0581 (17)	0.093 (2)	0.012 (2)	−0.059 (2)	−0.0290 (16)
C12	0.124 (2)	0.0733 (18)	0.120 (2)	0.0261 (16)	−0.0474 (18)	−0.0501 (16)
C13	0.0785 (14)	0.0644 (14)	0.0801 (14)	0.0057 (10)	−0.0166 (11)	−0.0346 (11)
C14	0.0465 (9)	0.0569 (11)	0.0612 (12)	−0.0039 (8)	−0.0003 (8)	−0.0196 (10)
C15	0.0543 (10)	0.0445 (10)	0.0564 (12)	−0.0065 (8)	−0.0046 (9)	−0.0130 (8)
C16	0.0563 (11)	0.0777 (14)	0.0644 (14)	−0.0013 (10)	−0.0035 (10)	−0.0220 (11)
C17	0.0973 (17)	0.0941 (17)	0.0563 (13)	−0.0017 (13)	−0.0136 (12)	−0.0184 (12)
C18	0.0826 (16)	0.0731 (15)	0.0820 (17)	0.0041 (11)	−0.0319 (13)	−0.0175 (12)
C19	0.0643 (12)	0.0576 (13)	0.0844 (16)	−0.0003 (9)	−0.0135 (11)	−0.0106 (11)
C20	0.0643 (12)	0.0464 (11)	0.0668 (12)	0.0001 (8)	−0.0058 (10)	−0.0067 (9)
C21	0.0422 (9)	0.0518 (10)	0.0590 (11)	−0.0001 (7)	−0.0071 (8)	−0.0221 (9)
C22	0.0568 (10)	0.0476 (10)	0.0518 (11)	−0.0031 (8)	−0.0138 (8)	−0.0196 (8)
C23	0.0550 (11)	0.0720 (13)	0.0673 (14)	−0.0009 (9)	−0.0164 (10)	−0.0205 (11)
C24	0.0903 (16)	0.0865 (16)	0.0689 (16)	0.0013 (12)	−0.0338 (13)	−0.0166 (13)
C25	0.1170 (19)	0.0876 (16)	0.0518 (12)	−0.0026 (14)	−0.0164 (14)	−0.0226 (11)
C26	0.0905 (15)	0.0952 (17)	0.0574 (14)	0.0033 (12)	−0.0041 (11)	−0.0286 (12)
C27	0.0699 (12)	0.0716 (13)	0.0551 (12)	0.0083 (9)	−0.0100 (10)	−0.0276 (10)

### Geometric parameters (Å, °)

**Table:**

N1—C6	1.4551 (19)	C12—C13	1.370 (3)
N1—C2	1.4567 (19)	C12—H12	0.9300
N1—C7	1.4612 (19)	C13—H13	0.9300
O1—C4	1.2223 (18)	C14—C15	1.465 (2)
F1—C16	1.357 (2)	C14—H14	0.9300
F2—C23	1.348 (2)	C15—C16	1.372 (3)
C2—C3	1.497 (2)	C15—C20	1.391 (2)
C2—H2A	0.9700	C16—C17	1.370 (3)
C2—H2B	0.9700	C17—C18	1.368 (3)
C3—C14	1.328 (2)	C17—H17	0.9300
C3—C4	1.501 (2)	C18—C19	1.374 (3)
C4—C5	1.491 (2)	C18—H18	0.9300
C5—C21	1.337 (2)	C19—C20	1.376 (2)
C5—C6	1.502 (2)	C19—H19	0.9300
C6—H6A	0.9700	C20—H20	0.9300
C6—H6B	0.9700	C21—C22	1.463 (2)
C7—C8	1.504 (2)	C21—H21	0.9300
C7—H7A	0.9700	C22—C23	1.386 (2)
C7—H7B	0.9700	C22—C27	1.388 (2)
C8—C9	1.370 (3)	C23—C24	1.366 (3)
C8—C13	1.375 (3)	C24—C25	1.367 (3)
C9—C10	1.402 (4)	C24—H24	0.9300
C9—H9	0.9300	C25—C26	1.374 (3)
C10—C11	1.358 (4)	C25—H25	0.9300
C10—H10	0.9300	C26—C27	1.375 (3)
C11—C12	1.353 (4)	C26—H26	0.9300
C11—H11	0.9300	C27—H27	0.9300
			
C6—N1—C2	108.20 (13)	C12—C13—C8	121.1 (2)
C6—N1—C7	111.83 (13)	C12—C13—H13	119.4
C2—N1—C7	111.85 (12)	C8—C13—H13	119.4
N1—C2—C3	109.17 (12)	C3—C14—C15	127.77 (15)
N1—C2—H2A	109.8	C3—C14—H14	116.1
C3—C2—H2A	109.8	C15—C14—H14	116.1
N1—C2—H2B	109.8	C16—C15—C20	115.62 (16)
C3—C2—H2B	109.8	C16—C15—C14	121.18 (16)
H2A—C2—H2B	108.3	C20—C15—C14	123.11 (17)
C14—C3—C2	124.42 (14)	F1—C16—C17	117.83 (19)
C14—C3—C4	118.45 (14)	F1—C16—C15	117.70 (16)
C2—C3—C4	117.12 (14)	C17—C16—C15	124.47 (19)
O1—C4—C5	121.80 (14)	C18—C17—C16	118.1 (2)
O1—C4—C3	121.17 (16)	C18—C17—H17	121.0
C5—C4—C3	116.93 (14)	C16—C17—H17	121.0
C21—C5—C4	117.70 (14)	C17—C18—C19	120.20 (19)
C21—C5—C6	125.03 (16)	C17—C18—H18	119.9
C4—C5—C6	117.28 (13)	C19—C18—H18	119.9
N1—C6—C5	110.11 (13)	C18—C19—C20	120.09 (19)
N1—C6—H6A	109.6	C18—C19—H19	120.0
C5—C6—H6A	109.6	C20—C19—H19	120.0
N1—C6—H6B	109.6	C19—C20—C15	121.52 (19)
C5—C6—H6B	109.6	C19—C20—H20	119.2
H6A—C6—H6B	108.2	C15—C20—H20	119.2
N1—C7—C8	112.87 (13)	C5—C21—C22	128.35 (16)
N1—C7—H7A	109.0	C5—C21—H21	115.8
C8—C7—H7A	109.0	C22—C21—H21	115.8
N1—C7—H7B	109.0	C23—C22—C27	115.28 (16)
C8—C7—H7B	109.0	C23—C22—C21	120.75 (17)
H7A—C7—H7B	107.8	C27—C22—C21	123.82 (15)
C9—C8—C13	118.18 (19)	F2—C23—C24	118.37 (17)
C9—C8—C7	120.25 (18)	F2—C23—C22	117.63 (17)
C13—C8—C7	121.45 (17)	C24—C23—C22	124.0 (2)
C8—C9—C10	120.5 (2)	C23—C24—C25	118.71 (19)
C8—C9—H9	119.8	C23—C24—H24	120.6
C10—C9—H9	119.8	C25—C24—H24	120.6
C11—C10—C9	119.6 (3)	C24—C25—C26	120.0 (2)
C11—C10—H10	120.2	C24—C25—H25	120.0
C9—C10—H10	120.2	C26—C25—H25	120.0
C12—C11—C10	120.1 (3)	C25—C26—C27	120.1 (2)
C12—C11—H11	119.9	C25—C26—H26	120.0
C10—C11—H11	119.9	C27—C26—H26	120.0
C11—C12—C13	120.5 (3)	C26—C27—C22	121.99 (18)
C11—C12—H12	119.8	C26—C27—H27	119.0
C13—C12—H12	119.8	C22—C27—H27	119.0
			
C6—N1—C2—C3	−69.01 (16)	C4—C3—C14—C15	175.34 (16)
C7—N1—C2—C3	167.39 (13)	C3—C14—C15—C16	147.49 (19)
N1—C2—C3—C14	−149.89 (16)	C3—C14—C15—C20	−36.2 (3)
N1—C2—C3—C4	28.8 (2)	C20—C15—C16—F1	−179.24 (16)
C14—C3—C4—O1	6.7 (2)	C14—C15—C16—F1	−2.6 (3)
C2—C3—C4—O1	−172.07 (15)	C20—C15—C16—C17	0.9 (3)
C14—C3—C4—C5	−169.87 (14)	C14—C15—C16—C17	177.48 (18)
C2—C3—C4—C5	11.4 (2)	F1—C16—C17—C18	179.90 (19)
O1—C4—C5—C21	−9.8 (2)	C15—C16—C17—C18	−0.2 (3)
C3—C4—C5—C21	166.67 (14)	C16—C17—C18—C19	−0.7 (3)
O1—C4—C5—C6	169.55 (15)	C17—C18—C19—C20	0.9 (3)
C3—C4—C5—C6	−13.9 (2)	C18—C19—C20—C15	−0.2 (3)
C2—N1—C6—C5	66.60 (16)	C16—C15—C20—C19	−0.7 (3)
C7—N1—C6—C5	−169.78 (12)	C14—C15—C20—C19	−177.20 (17)
C21—C5—C6—N1	155.39 (15)	C4—C5—C21—C22	−172.85 (15)
C4—C5—C6—N1	−23.94 (19)	C6—C5—C21—C22	7.8 (3)
C6—N1—C7—C8	75.65 (17)	C5—C21—C22—C23	−151.13 (17)
C2—N1—C7—C8	−162.82 (14)	C5—C21—C22—C27	33.5 (3)
N1—C7—C8—C9	−153.28 (15)	C27—C22—C23—F2	−179.31 (16)
N1—C7—C8—C13	30.8 (2)	C21—C22—C23—F2	5.0 (3)
C13—C8—C9—C10	0.8 (3)	C27—C22—C23—C24	0.1 (3)
C7—C8—C9—C10	−175.21 (17)	C21—C22—C23—C24	−175.57 (17)
C8—C9—C10—C11	−0.7 (3)	F2—C23—C24—C25	179.65 (19)
C9—C10—C11—C12	0.2 (4)	C22—C23—C24—C25	0.2 (3)
C10—C11—C12—C13	0.1 (4)	C23—C24—C25—C26	−0.5 (3)
C11—C12—C13—C8	0.1 (3)	C24—C25—C26—C27	0.5 (3)
C9—C8—C13—C12	−0.5 (3)	C25—C26—C27—C22	−0.2 (3)
C7—C8—C13—C12	175.46 (17)	C23—C22—C27—C26	−0.2 (3)
C2—C3—C14—C15	−6.0 (3)	C21—C22—C27—C26	175.40 (18)

### Hydrogen-bond geometry (Å, °)

**Table:**

*D*—H···*A*	*D*—H	H···*A*	*D*···*A*	*D*—H···*A*
C14—H14···O1	0.93	2.40	2.772 (2)	104
C21—H21···O1	0.93	2.39	2.768 (2)	104
C7—H7B···Cg2^i^	0.97	2.78	3.7315 (19)	168
C13—H13···N1	0.93	2.56	2.873 (3)	100

Symmetry codes: (i) −*x*+1, −*y*+2, −*z*+2.
